# Effectiveness of a structured short intervention against stigmatisation in chronic visible skin diseases: Results of a controlled trial in future educators

**DOI:** 10.1111/hex.13319

**Published:** 2021-07-27

**Authors:** Natascha‐Alexandra Weinberger, Sonja Mrowietz, Claudia Luck‐Sikorski, Regina von Spreckelsen, Sven M. John, Rachel Sommer, Matthias Augustin, Ulrich Mrowietz

**Affiliations:** ^1^ Research Group: Chronic Diseases and Psychological Health (COPE) University of Applied Health Sciences SRH Gera Gera Germany; ^2^ Vocational College for Social Pedagogy RBZ Koenigsweg Kiel Germany; ^3^ Psoriasis‐Center at the Department of Dermatology University Medical Center Schleswig‐Holstein, Campus Kiel Kiel Germany; ^4^ Department of Dermatology, Environmental Medicine, Health Theory, Institute for Interdisciplinary Dermatological Prevention and Rehabilitation (iDerm) University of Osnabrueck Osnabrueck Germany; ^5^ Institute for Health Services Research in Dermatology and Nursing (IVDP), German Center for Health Services Research in Dermatology (CVderm) University Medical Center Hamburg‐Eppendorf (UKE) Hamburg Germany

**Keywords:** adults, dermatology, intervention, psoriasis, stigmatisation

## Abstract

**Background:**

Chronic visible skin diseases are highly prevalent, and patients affected frequently report feeling stigmatised. Interventions to reduce stigmatisation are rare.

**Objectives:**

This study aimed to evaluate the effectiveness of a structured short intervention in reducing stigmatising attitudes towards psoriasis in future educators.

**Methods:**

The intervention consisted of four components: (1) self‐reflection, (2) education on skin diseases, (3) contact between participants and a person with psoriasis and (4) practising of knowledge via case studies. A quasi‐experimental, pre–post study design was chosen with a nonrandomized contemporaneous control group that attended regular lessons. The main outcomes were participants' desire for social distance, stereotype endorsement, illness‐related misconceptions and intended behaviour. Intervention effects were analysed using mixed repeated‐measures analysis of variance, with Bonferroni post‐hoc tests for pairwise comparisons.

**Results:**

The sample consisted of 221 students attending vocational training as educators (*n* = 118 intervention group, *n* = 103 control group). While no effect of the intervention was found in social distance, small to large effect sizes were observed for intended behaviour (*r* = .14), illness‐related misconceptions (*r* = .28) and stereotype endorsement (*r* = .42). The intervention group reported significantly higher satisfaction with the seminar compared to the control group.

**Conclusions:**

Overall, the short intervention was effective at reducing stigmatising attitudes in future educators. In perspective, revised versions could help in reducing stigmatisation in various demographics and promote patient empowerment by acknowledging and including them as experts on their own behalf.

**Patient or Public Contribution:**

Patient advocate groups were consulted and involved in the superordinate destigmatization research programme and intervention.

## 1 BACKGROUND AND OBJECTIVES

Chronic visible skin diseases are a highly prevalent health problem. In Germany, around 10 million people are affected, most commonly by psoriasis, acne, rosacea and atopic dermatitis.^1^, ^2^ In addition to medical consequences, patients have a substantial psychosocial burden. A study among dermatological outpatients in 13 European countries^3^ reported the presence of depression in 10.1%, anxiety in 17.2% and suicidal ideation in 12.7% of all patients. Patients with skin conditions also frequently report an impaired health‐related quality of life^4^ and feelings of stigmatisation.^5^, ^6^

Goffmann^7^ (p. 3) defined stigma as a ‘deeply discrediting attribute’ that reduces the stigmatised individual ‘from a whole and usual person to a tainted, discounted one’. Over time, the definition was extended and adapted, but essential components of stigma are generally agreed to be the recognition of a difference and devaluation and that stigma is a social construct.^8^ Link and Phelan^9^ broadened previous stigma concepts by including the perspective of the stigmatised and highlighting the influence of sociocultural processes in stigmatisation. According to their conceptualisation, stigma exists when four interrelated components converge: Individuals identify a difference and ‘put a label’ on it. Subsequently, (negative) stereotypes are linked to labelled individuals and they are placed in distinct categories to establish a sense of separation between ‘us’ and ‘them’. Finally, labelled individuals experience status loss and discrimination that leads to unequal circumstances. The occurrence and associated negative consequences of stigmatisation have been reported in previous studies with regard to numerous illnesses like human immunodeficiency virus/acquired immunodeficiency syndrome, mental illness and obesity.^10^, ^11^, ^12^ A substantial number of studies on the topic of stigmatisation in skin diseases so far have focused on psoriasis^5^ and the World Health Assembly declared it to be a noncommunicable disease with particular relevance for health care delivery in 2014.^13^

The visibility of skin changes in psoriasis facilitates the identification and labelling of a difference. Stereotypes about issues such as inadequate body hygiene lead to attribution of responsibility for the disease, while fear of infection or disgust results in a desire for social distance. Misconceptions about psoriasis and stigmatising attitudes are frequent in the general public. Halioua et al.^14^ report that 16.5% of respondents believe that psoriasis is contagious and 6.8% believe that skin disease is related to personal hygiene. About half of the sample showed discriminatory behaviour towards patients with psoriasis, for example, reluctance to maintain friendship ties, to have a meal with a person with visible skin changes or to shake hands. Moreover, 8% of respondents reported negative feelings about psoriasis, such as disgust, fear and rejection. Similarly, a representative survey investigating the perception of psoriasis among the German population found that 9% of respondents considered the disease to be communicable, 13% of the respondents stated that they would not shake hands and 7% of respondents would not want to eat at the same table with a person with psoriasis. A total of 10% reported that they would not want to live in the same household as an affected individual, 23% did not want to share the same swimming pool and 27% did not want to be in a personal relationship with someone affected by the disease.^15^

For individuals affected by psoriasis, internalised stigma is frequent.^16^ In addition to (self‐) stigmatisation, previous studies also report a high perceived burden of social isolation based on the fear of social rejection, and impaired body image in affected individuals, all of which were associated with depressive symptoms.^17^ In line with these findings, stigmatisation in psoriasis has been linked to impaired quality of life and mental health.^18^ In psoriasis patients, stigmatisation was even found to be the most powerful predictor of depressive symptoms.^19^ Consequently, affected individuals face a double burden of disease: the dermatological diagnosis itself and its potential stigmatisation.

It is therefore necessary to increase awareness and take steps in increasing acceptance and reducing stigmatisation of skin diseases as urged by recent World Health Organization recommendations.^20^ There is clear consensus among health care professionals, researchers and patient organisations that systematic action is needed to, for example, raise awareness among the general public that psoriasis is a chronic condition that is not contagious, to identify and adequately treat patients who might be particularly vulnerable to stigmatisation and its negative consequences and to develop interventions that target the societal stigmatisation and discrimination of psoriasis.^21^, ^22^, ^23^

Link and Phelan^9^ (p. 367) specified that the term stigma can be applied ‘when elements of labelling, stereotyping, separation, status loss, and discrimination co‐occur in a power situation that allows the components of stigma to unfold’. It is therefore important for intervention efforts to address (potential) community facilitators in positions of influence, to reduce their stigmatising attitudes as early as possible and subsequently turn their societal or professional position to account. Individuals or groups that are frequently considered to be authority figures could for example contribute towards spreading awareness, correcting common misconceptions and preventing discrimination of individuals with skin diseases in future interactions. Moreover, patients are confronted with stigmatisation not just in adulthood but also already from childhood and adolescence.^24^

The current short intervention was therefore focused on preschool teachers in training at a vocational college in Germany to take advantage of their unique position in not only educating children from a very young age but also being in frequent contact with parents of different societal and professional backgrounds. The development of the current intervention was based on both practical considerations regarding implementation, future adaptability to different contexts and target groups and past evidence.

The primary objective of this study was to evaluate how effective the developed short intervention would be in reducing stigmatising attitudes in the participants of the intervention group in comparison to the control group. Ascertaining the predictive value of group status as well as confounding variables with regard to intervention effects were key secondary objectives. Another secondary objective was to investigate participants' satisfaction with the intervention to identify aspects that were viewed (un‐)favourably, so future versions of the seminar can be modified accordingly.

## 2 METHODS

### 2.1 Trial design

The methodological approach of the superordinate destigmatization research programme and intervention has been detailed before.^25^ In addition to different professions working in research and treatment, patient advocate groups were consulted and involved in the development and long‐term implementation of the overarching research programme as well as the communication of its activities and results. Moreover, patients with psoriasis were directly involved in the short intervention as experts on their own behalf in the trialogue with participants and health professional.

Evaluation of the short intervention followed a nonrandomized quasi‐experimental, pre–post study design with a contemporaneous control group. Due to the structure of the vocational education, assignment to study and control groups had to be done on the class level to avoid multiple or repeated participation by students of the same academic year. Participants in the control and intervention group participated in the study in parallel and were surveyed three times: immediately before (*t*_0_) and after the intervention/regular class (*t*_1_), and at 3 months of follow‐up (*t*_2_) to ascertain medium‐term effects.

### 2.2 Participants

The final sample consisted of *N* = 221 students (*n* = 118 intervention group, *n* = 103 control group) attending vocational training as educators at a college of social pedagogy in the city of Kiel, Germany. Students eligible for participation had to be older than 18 years of age. Participants provided written informed consent and received €100 for their class fund at the end of the study. The study was approved by the local institutional review board (AZ: D521‐18) and the Ministry of Education of the local government of Schleswig‐Holstein. Sociodemographic data of the sample as well as statistical differences between the control and intervention groups are presented in Table 1.

**Table 1:** Descriptive statistics (mean and standard deviation) of sociodemographic data, depressive symptoms, personal affliction with a skin disease, contact with people affected by a skin disease and stigmatising attitudes at *t*_0_

	Total	Intervention	Control	Difference
	(*N* = 221)	(*n* = 118)	(*n* = 103)	
	*M* (*SD*)	*M* (*SD*)	*M* (*SD*)	
Sex	1.75 (0.44)	1.74 (0.44)	1.76 (0.43)	*χ*^2^(1) = 0.12
*n* (%) Female	*165 (74.7)*	*78 (73.7)*	*78 (75.7)*	
*n* (%) Male	*56 (25.3)*	*31 (26.3)*	*25 (24.3)*	
Age^a^	23.83 (5.36)	23.58 (5.44)	24.13 (5.28)	*t*(216) = −0.76
Prev. vocational training	0.61 (0.49)	0.62 (0.49)	0.61 (0.49)	*χ*^2^(1) = 0.13
*n* (%) Yes	*134 (61.2)*	*72 (61.5)*	*62 (60.8)*	
*n* (%) No	*85 (38.8)*	*45 (38.5)*	*40 (39.2)*	
Affected by skin disease^b^	0.14 (0.35)	0.17 (0.38)	0.10 (0.30)	*χ*^2^(1) = 2.36
*n* (%) Yes	*30 (13.8)*	*20 (17.1)*	*10 (9.9)*	
*n* (%) No	*188 (86.2)*	*97 (82.9)*	*91 (90.1)*	
Depressive symptoms^c^	6.43 (4.29)	6.17 (3.84)	6.73 (4.76)	*t*(217) = −0.95
*n* (%) Scores < *5*	*82 (37.6)*	*48 (41.0)*	*34 (33.7)*	
*n* (%) Scores > *5*	*136 (62.4)*	*69 (59.0)*	*67 (66.3)*	
Patient contact^d^	1.98 (0.81)	2.05 (0.88)	1.90 (0.71)	*z* = −1.02
Stereotype endorsement	2.81 (0.63)	2.80 (0.63)	2.82 (0.64)	*t*(215) = −0.33
Social distance	1.78 (0.70)	1.80 (0.71)	1.76 (0.68)	*t*(217) = 0.55
Illness misconceptions	1.77 (0.63)	1.74 (0.66)	1.79 (0.59)	*t*(211) = −0.59
Intended behaviour	17.62 (2.86)	17.55 (2.96)	17.71 (2.76)	*t*(215) = −0.42

*Note:* The italic values illustrate the distribution of responses.

^a^ *n* = 3 Subjects reported 2019 as their year of birth.

^b^ Subjects reported if they were personally affected by a skin disease.

^c^ Scores below 5 indicate a lack of depressive symptoms.

^d^ Subjects reported how often they had contact with persons affected by skin disease in their daily life, ranging from 1 = never to 5 = very often.

### 2.3 Intervention

The intervention consisted of four components: (1) self‐reflection and discourse about their role in and their experience with stigmatisation between the participants of the seminar, (2) education on skin diseases and common misconceptions as well as the process of stigmatisation, (3) an encounter between participants and a patient affected by a skin disease under the supervision of a health professional and (4) development and discussion of varying courses of action when confronted with stigmatising situations by means of case studies. The intervention was developed to fit the usual length and structure of the regular classes of the participating college to ensure blinding of the study groups to the greatest possible extent. To control for the potential role of disease pattern and/or specific symptoms that might influence stigmatisation, the intervention and evaluation were geared towards the disease of psoriasis. In particular, the same patient (male, 33 years old, affected by psoriasis since childhood, currently undergoing infusion treatment) participated in all interventions. The intervention was conducted by a lecturer of the vocational colleague in cooperation with a dermatologist who moderated the exchange between participants and patients and was readily available to answer medical questions. Both had taken part in its development and as such were familiar with the seminar's structure and content. A detailed overview of the intervention can be found in the appendix. The control group attended a regular class of their vocational training during the same period.

### 2.4 Outcomes

The primary endpoint with respect to the effectiveness of the intervention was the reduction of stigmatising attitudes in participants in the intervention group in comparison to the control group from baseline to *t*_1_, with scores remaining consistently low at follow‐up as measured by the stereotype endorsement scale, the social distance scale, the psoriasis myth endorsement scale and the reported and intended behaviour scale (see below for details). Secondary analyses were done to ascertain the role of group status as well as confounding variables (see below for details), and to analyse satisfaction of participants with the seminar.

#### 2.4.1 Primary outcome measures

For their study of stigmatising attitudes towards persons with psoriasis in laypersons and medical students, Pearl et al.^26^ adapted and developed instruments to assess stigmatisation in cooperation with dermatologists with expertise in psoriasis. The current study adapted the scales for use in German samples with their permission and undertook an initial validation during a pilot study. Two of the original scales (‘Emotions’, ‘Attitudes toward treating patients’) were not included due to time constraints and irrelevance to the current sample, respectively.

##### 2.4.1.1 Stereotype endorsement

A semantic differential scale consisting of 11 adjective pairs (e.g., unattractive–attractive) was used to assess stereotype endorsement of individuals with psoriasis. Participants were asked to mark the circle closest to the adjective that they considered to describe a person with psoriasis (ranging from 1 to 5, with 1 being closest to the positive adjective). Scores were averaged, with higher scores indicating greater endorsement of negative stereotypes.

##### 2.4.1.2 Social distance

The social distance scale measures a participant's desire to eschew persons with psoriasis in nine different social situations (e.g., ‘share a meal’). Items were rated on a 5‐point Likert scale ranging from ‘definitely’ to ‘definitely not’. Item scores were averaged, with lower scores indicating lower desire for social distance.

##### 2.4.1.3 Illness‐related misconceptions (‘myth endorsement’)

Agreement with common misconceptions about the disease was analysed with 15 statements about psoriasis (e.g., ‘psoriasis is caused by poor hygiene’). Participants rated the statements on a 5‐point Likert scale ranging from ‘strongly disagree’ to ‘strongly agree’. Scores were averaged, with lower scores indicating lower endorsement of the misconceptions.

##### 2.4.1.4 Reported and Intended Behaviour Scale

The Reported and Intended Behaviour Scale (RIBS)^27^ was adapted for use in skin diseases to explore stigmatising behaviour in the current study. Four items of the RIBS assess the prevalence of behaviour in each of four contexts (living with, working with, living nearby and continuing a relationship with someone affected by psoriasis), while four more items assess intended behaviour within the same contexts. The items assessing prevalence of behaviour followed a dichotomous response format (yes–no), while items assessing intended behaviour were scored on a 5‐point Likert scale ranging from ‘strongly disagree’ to ‘strongly agree’. The total score of intended behaviour was the sum of response values, with higher scores indicating less stigmatising behaviour.

#### 2.4.2 Secondary outcome measures/confounder

Participants reported sociodemographic data (age, gender and previous vocational training in a social field), as well as how often they had contact with individuals affected by skin diseases in their daily lives and whether they were affected by a skin disease themselves at the beginning of the survey (see Table 1). In addition, participants were screened for depressive symptoms to explore their potential influence on their stigmatising attitudes and their evaluation of the attended seminar.

##### 2.4.2.1 Patient Health Questionnaire‐9

The depression module of the Patient Health Questionnaire (PHQ) is a valid and reliable self‐report measure to assess depressive symptoms.^28^ Participants were asked to report for nine depressive symptoms, respectively, in terms of whether and how often these had bothered them in the previous 2 weeks. The total score—a measure of depression severity—ranges between 0 and 27, with scores of 5, 10, 15 and more indicating mild, moderate and moderate to severe symptoms of depression.

##### 2.4.2.2 Satisfaction with the seminar

A short self‐developed questionnaire was used at data collection point *t*_1_ and *t*_2_ to measure participants' satisfaction with the (intervention) class. The 12 items were selected to ascertain for example, students' satisfaction with the length and content of the seminar, personal estimation of its relevancy in their (working) life and whether they would recommend the seminar to other students. Participants rated the statements on a 5‐point Likert scale ranging from ‘strongly disagree’ to ‘strongly agree’. Scores were averaged, with higher scores indicating higher satisfaction.

In addition, a free‐text feedback box that allowed general comments and suggestions was displayed at the very end of the questionnaire.

### 2.5 Sample size calculation

Due to the lack of intervention studies addressing stigma in this field, the results of a previously conducted pilot study were used for an estimation of effects. An a priori power analysis was conducted using G*Power. With an alpha level of .05, minimum power established at .90 and a moderate expected effect size of .45,^29^ 210 participants would be necessary to find a statistically significant effect in the model.

### 2.6 Group allocation

As was indicated previously, the allocation of students to the control and intervention groups took place on the class level. To decrease the risk of bias, group assignment was decided by the school timetable of September 2019 in combination with the availability of the psoriasis patient and the medical expert, respectively. Class hours were compared to dates and time given by the patient and medical expert, and classes matching these dates were allocated to the intervention group, while classes not matching the dates were allocated to the control group.

### 2.7 Statistical methods

Data analyses were carried out using SPSS 25.0.0.^30^ *T* tests, Mann–Whitney *U* tests and *χ* ^2^ tests were used to analyse differences between groups in sociodemographic as well as the main outcome variables (see Table 1).

Intervention effects were analysed using mixed repeated‐measures analysis of variance, with Bonferroni post‐hoc tests for pairwise comparisons. Due to violation of the assumption of a normal distribution in social distance scores, a Friedman test was used to analyse the differences between the study and control groups in this outcome, with Wilcoxon signed‐rank tests with Bonferroni correction aiding in post‐hoc analysis.

Multiple regression analyses were conducted to examine whether the four main outcome scores at *t*_1_ and *t*_2,_ respectively, can be predicted based on study group membership, gender, age, depressive symptoms, personal affliction with a skin disease, frequency of contact with individuals with skin diseases and previous vocational training in a social field (see Tables 2, 3, 4, 5).

**Table 2:** Regression analysis predicting post‐intervention scores for stereotype endorsement

Variable	*t*_1_			*t*_2_		
	*B*	*SE B*	Beta (*β*)	*B*	*SE B*	Beta (*β*)
Constant	2.939	0.260		2.924	0.292	
Group	−0.164	0.081	−.134*	−0.143	0.090	−.117
Gender	−0.307	0.092	−.220**	−0.259	0.100	−.190*
Age	0.006	0.008	.053	0.004	0.009	.031
Depressive symptoms	0.004	0.009	.032	0.003	0.010	.022
Affected by skin disease	−0.327	0.117	−.190**	−0.077	0.132	−.045
Vocational training	−0.116	0.043	−.179**	−0.179	0.048	−.280***
Patient contact	−0.035	0.051	−.046	0.012	0.055	.016

*Note:* Outcome variable: stereotype endorsement (1 = positive adjective to 5 = negative adjective).^26^ Predictor variables: group (0 = control group, 1 = intervention group), gender (0 = male, 1 = female), age, depressive symptoms (PHQ sum‐score), personal affliction with skin disease (0 = no, 1 = yes), previous vocational training (0 = no, 1 = yes) and contact with individuals affected by skin disease in daily live (1 = never, 5 = very often).

Model fit calculated from valid cases: (*t*_1_) *F*(7, 199) = 5.21, *p* < .001, adjusted *R* ^2^ = .13. (*t*_2_) *F*(7, 164) = 3.98, *p* < .001, adjusted *R* ^2^ = .11.

Abbreviations: *β*, standardised regression coefficient; *B*, unstandardised regression coefficient; *SE*, standard error.

* *p* < .05

** *p* < .01

*** *p* < .001.

**Table 3:** Regression analysis predicting postintervention scores for social distance

Variable	*t*_1_			*t*_2_		
	*B*	*SE B*	Beta (*β*)	*B*	*SE B*	Beta (*β*)
Constant	1.742	0.302		2.288	0.315	
Group	−0.158	0.093	−.117	−0.005	0.097	−.004
Gender	0.023	0.107	.015	−0.012	0.108	−.008
Age	0.007	0.009	.057	−0.011	0.010	−.089
Depressive symptoms	0.006	0.011	.040	0.004	0.011	.032
Affected by skin disease	−0.330	0.138	−.170*	−0.196	0.146	−.106
Vocational training	−0.060	0.050	−.084	−0.084	0.052	−.125
Patient contact	−0.092	0.059	−.109	−0.129	0.060	−.165*

*Note:* Outcome variable: social distance (1 = definitely to 5 = definitely not).^26^ Predictor variables: group (0 = control group, 1 = intervention group), gender (0 = male, 1 = female), age, depressive symptoms (PHQ sum‐score), personal affliction with skin disease (0 = no, 1 = yes), previous vocational training (0 = no, 1 = yes) and contact with individuals affected by skin disease in daily live (1 = never, 5 = very often).

Model fit calculated from valid cases: (*t*_1_) *F*(7, 199) = 2.42, *p* = .021, adjusted *R* ^2^ = .05. (*t*_2_) *F*(7, 166) = 1.78, *p* = .094, adjusted *R* ^2^ = .03.

Abbreviations: *β*, standardised regression coefficient; *B*, unstandardised regression coefficient; *SE*, standard error.

* *p* < .05.

**Table 4:** Regression analysis predicting postintervention scores for illness‐related misconceptions

Variable	*t*_1_			*t*_2_		
	*B*	*SE B*	Beta (*β*)	*B*	*SE B*	Beta (*β*)
Constant	1.990	0.215		2.146	0.301	
Group	−0.382	0.067	−.371***	−0.337	0.093	−.271***
Gender	−0.131	0.077	−.111	−0.016	0.104	−.011
Age	−0.004	0.006	−.043	−0.002	0.009	−.017
Depressive symptoms	0.003	0.008	.029	−0.012	0.010	−.089
Affected by skin disease	−0.167	0.098	−.114	−0.171	0.138	−.096
Vocational training	−0.027	0.036	−.050	−0.071	0.050	−.107
Patient contact	−0.009	0.042	−.015	−0.054	0.058	−.070

*Note:* Outcome variable: illness‐related misconceptions (1 = strongly disagree to 5 = strongly agree).^26^ Predictor variables: group (0 = control group, 1 = intervention group), gender (0 = male, 1 = female), age, depressive symptoms (PHQ sum‐score), personal affliction with skin disease (0 = no, 1 = yes), previous vocational training (0 = no, 1 = yes) and contact with individuals affected by skin disease in daily live (1 = never, 5 = very often).

Model fit calculated from valid cases: (*t*_1_) *F*(7, 200) = 6.23, *p* < .001, adjusted *R* ^2^ = .15. (*t*_2_) *F*(7, 165) = 2.95, *p* = .006, adjusted *R* ^2^ = .07.

Abbreviations: *β*, standardised regression coefficient; *B*, unstandardised regression coefficient; *SE*, standard error.

*** *p* < .001.

**Table 5:** Regression analysis predicting postintervention scores for intended behaviour

Variable	*t*_1_			*t*_2_		
	*B*	*SE B*	Beta (*β*)	*B*	*SE B*	Beta (*β*)
Constant	16.968	1.174		15.563	1.474	
Group	0.395	0.363	.074	−0.122	0.456	−.021
Gender	0.479	0.415	.079	0.958	0.506	.146
Age	−0.009	0.035	−.019	0.000	0.045	.000
Depressive symptoms	−0.090	0.042	−.147*	0.022	0.050	.035
Affected by skin disease	1.221	0.531	.161*	1.013	0.676	.120
Vocational training	0.082	0.195	.029	0.030	0.242	.010
Patient contact	0.414	0.228	.125	0.494	0.280	.138

*Note:* Outcome variable: intended behaviour (RIBS; 1 = strongly disagree to 5 = strongly agree) 27. Predictor variables: group (0 = control group, 1 = intervention group), gender (0 = male, 1 = female), age, depressive symptoms (PHQ sum‐score), personal affliction with skin disease (0 = no, 1 = yes), previous vocational training (0 = no, 1 = yes) and contact with individuals affected by skin disease in daily live (1 = never, 5 = very often).

Model fit calculated from valid cases: (*t*_1_) *F*(7, 201) = 2.94, *p* = .006, adjusted *R* ^2^ = .06. (*t*_2_) *F*(7, 165) = 1.37 and *p* = .221, adjusted *R* ^2^ = .02.

Abbreviations: *β*, standardised regression coefficient; *B*, unstandardised regression coefficient; *SE*, standard error.

* *p* < .05.

*p*‐Values were obtained from two‐tailed tests, with a *p* < .05 indicating statistical significance.

## 3 RESULTS

### 3.1 Participant flow and recruitment

Overall, 253 students filled in questionnaires at the three data collection points within their classes. Three students did not fill out the *t*_0_ questionnaire, *n* = 11 did not report data for the *t*_1_ questionnaire and *n* = 18 students reported data for the *t*_2_ questionnaire only, leaving 221 questionnaires for statistical analysis. In addition, *n* = 38 students did not participate in the follow‐up survey *t*_2._

Eligible students attended either the intervention or a vocational class in September 2019; follow‐up surveys took place 12 weeks later in November and December 2019.

### 3.2 Baseline data

The control and intervention groups did not differ significantly regarding their sociodemographic data or the main outcome measures at *t*_0_. Descriptive statistics for the total sample as well as the control and intervention groups separately are illustrated in Table 1.

### 3.3 Numbers analysed

Analyses included all participants who filled in both the *t*_0_ and *t*_1_ questionnaires (*n* = 221). A total of 21 participants of the control group and 17 participants of the intervention group were lost to follow‐up *t*_2_; thus, data from 183 students were available for primary analysis.

### 3.4 Primary outcomes

#### 3.4.1 Stigmatising attitudes towards individuals with psoriasis

Over the survey period, no interaction effect between study group and time was determined for stereotype endorsement (Greenhouse–Geisser *F*(1.82, 320.08) = 1.26, *p* = .283, partial *η*²* *= 0.01). Stereotype endorsement was significantly reduced over time in both the control group (Greenhouse–Geisser *F*(1.74, 137.47) = 8.09, *p* = .001, partial *η*² = 0.09) and the intervention group (Greenhouse–Geisser *F*(1.66, 161.42) = 20.72, *p* < .001, partial *η*² = 0.18). Stereotype endorsement scores in the intervention group differed significantly between *t*_0_–*t*_1_ (0.33, *p* < .001) and *t*_0_–*t*_2_ (0.29, *p* < .001), while no significant difference was found between *t*_1_ and *t*_2_ (−0.04, *p* = .925). In the control group, scores differed significantly between *t*_0_–*t*_1_ (0.21, *p* < .001) and *t*_0_–*t*_2_ (0.20, *p* = .014), but not between *t*_1_ and *t*_2_ (−0.01, *p* = 1.00). In addition, a significant group effect was observed (*F*(1, 176)* *= 4.19, *p* = .042, partial *η*² = 0.02). While stereotype endorsement in the intervention and control groups did not differ at *t*_0_ (*t*(179) = −0.84, *p* = .403) and *t*_2_ (*t*(178) = −1.84, *p* = .067), scores in the intervention group were significantly lower compared to the control group at *t*_1_ (*t*(179) = −2.46, *p* = .015).

Further, desire for social distance was significantly reduced in the intervention group (*χ* ^2^(2) = 33.87, *p* < .001). Post‐hoc analysis found significant differences between the time points *t*_0_–*t*_1_ (*Z* = 0.76, *p* < .001; *d* = 0.15) and *t*_0_–*t*_2_ (*Z* = 0.44, *p* = .006; *d* = 0.09). No significant difference in scores was found between *t*_1_ and *t*_2_ (*Z* = −0.32, *p* = .076). In the control group, no significant change in social distance scores over time could be observed (*χ* ^2^(2) = 3.80, *p* = .150). Social distance scores in the control and study groups did not differ significantly at any data collection point (*t*_0_ *U* = 3849.50, *Z* = −0.82, *p* = .412; *t*_1_ *U* = 3475.50, *Z* = −1.78, *p* = .074; *t*_2_ *U* = 4005.00, *Z* = −0.27, *p* = .787).

For illness‐related misconceptions, a significant interaction between study group and time was observed (Greenhouse–Geisser *F*(1.90, 324.95) = 14.41, *p* < .001, partial *η*² = 0.08). While no significant change in illness‐related misconception scores was found in the control group (Greenhouse–Geisser *F*(1.74, 135.45) = 0.17, *p* = .813, partial *η*² = 0.00), a significant reduction in scores over time was found in the intervention group (Greenhouse–Geisser *F*(1.90, 176.33) = 30.51, *p* < .001, partial *η*² = 0.25). Scores in the intervention group differed significantly between *t*_0_–*t*_1_ (0.40, *p* < .001) and *t*_0_–*t*_2_ (0.35, *p* < .001). No significant difference was observed between *t*_1_ and *t*_2_ (−0.05, *p* = .932). Moreover, a significant effect of study group was observed in this outcome (*F*(1, 171) = 17.01, *p* < .001, partial *η*² = 0.09). While the control and intervention groups did not significantly differ in illness‐related misconceptions at *t*_0_ (*t*(176) = −0.57, *p* = .569), endorsement scores were significantly lower in the intervention group compared to the control group at *t*_1_ (*t*(178) = −6.38, *p* < .001) and *t*_2_ (*t*(179) = −4.76, *p* < .001).

Further, a significant interaction effect between study group and time was observed for intended behaviour (Greenhouse–Geisser *F*(1.90, 335.46) = 4.40, *p* = .015, partial *η*² = 0.02).

Intended behaviour scores significantly changed over time in the intervention group (Greenhouse–Geisser *F*(1.84, 179.80) = 4.86, *p* = .011, partial *η*² = 0.05), but not in the control group (Greenhouse–Geisser *F*(1.94, 152.99) = 1.08, *p* = .340, partial *η*² = 0.01).

In the intervention group, scores differed significantly between *t*_0_ and *t*_1_ (−0.76, *p* = .004), while no significant differences were found between *t*_1_–*t*_2_ (0.58, *p* = .052) and *t*_0_–*t*_2_ (−0.18, *p* = 1.00). The group effect for this outcome also did not reach significance (*F*(1, 177) = 0.64, *p* = .424, partial *η*² = 0.00). The intervention group reported higher intended behaviour scores compared to the control group only at *t*_1_ (*t*(181) = 2.22, *p* = .028).

### 3.5 Secondary outcomes

#### 3.5.1 Role of group status

All variables and their statistical contribution to the prediction of the main outcome scores at *t*_1_ and *t*_2_ are illustrated in Tables 2, 3, 4, 5.

Participants' study group status, gender, age, depressive symptoms, frequency of contact with individuals affected by a skin disease, personal affliction with a skin disease and previous vocational training in a social field explained a significant proportion of variance in social distance at *t*_1_ (*R* ^2^ = .05, *F*(7, 199) = 2.42, *p* = .021), but not at *t*_2_ (*R* ^2^ = .03, *F*(7, 166) = 1.78, *p* = .094). At both time points, group status did not contribute significantly to the prediction of social distance.

Further, the predictor variables explained a significant proportion of variance in stereotype endorsement at *t*_1_ (*R* ^2^ = .13, *F*(7, 199) = 5.21, *p* < .001) and at *t*_2_ (*R* ^2^ = .11, *F*(7, 164) = 3.98, *p* < .001). For both time points, group status as well as gender and previous vocational training contributed significantly to the prediction. In addition, personal affliction with a skin disease was a significant contributor to explain stereotype endorsement scores at *t*_1_.

Moreover, the predictor variables explained a significant proportion of variance in illness‐related misconceptions at *t*_1_ (*R* ^2^ = .15, *F*(7, 200) = 6.23, *p* < .001) and *t*_2_ (*R* ^2^ = .07, *F*(7, 165) = 2.95, *p* = .006). Group status was the only significant contributor at both time points for this outcome.

Finally, the predictor variables explained a significant proportion of variance in intended behaviour at *t*_1_ (*R* ^2^ = .06, *F*(7, 201) = 2.94, *p* = .006), but not at *t*_2_ (*R* ^2^ = .02, *F*(7, 165) = 1.37, *p* = .221). Two significant contributors were observed: Depressive symptoms and personal affliction with a skin disease.

#### 3.5.2 Evaluation of the attended class

In comparison to the control group, participants in the intervention group were more satisfied with the class they had attended immediately after it ended (*t*(181) = 10.28, *p* < .001), as well as 3 months later (*t*(180) = 8.89, *p* < .001). Thirty‐three participants filled in the free‐text feedback box at the end of the questionnaire. The most frequent comments thanked the organisers (*n* = 15), suggested spending even more time with the patient (*n* = 5) and declared interest in future seminars including different skin diseases or more than one patient (*n* = 4).

## 4 DISCUSSION

The current study aimed to evaluate the effectiveness of the developed short intervention in reducing stigmatising attitudes in its participants. In addition, the role of group status as well as confounding variables regarding intervention effects was analysed. Participants' satisfaction with the attended intervention was investigated.

Participants in the intervention group reported significantly lower scores at follow‐up for three of the four analysed outcome variables (stereotype endorsement, illness‐related misconceptions and social distance). Scores for the fourth outcome, intended behaviour, increased immediately after the intervention, but had decreased to resemble the initial values at follow‐up. The intervention and control groups differed significantly in their scores immediately after the seminar in three of the four outcome variables (stereotype endorsement, illness‐related misconceptions and intended behaviour). At follow‐up, the two groups only differed significantly in illness‐related misconceptions. While no effect of the intervention was found in social distance, small to large effect sizes could be observed for intended behaviour, illness‐related misconceptions and stereotype endorsement.

The group status was a significant predictor in stereotype endorsement and illness‐related misconceptions, but not in social distance and intended behaviour.

Participants in the intervention group reported significantly higher satisfaction with the class that they had attended immediately after and at follow‐up in comparison to the control group.

The current intervention included two of the four approaches (contact with patients affected by a skin disease and education on skin diseases and common misconceptions) identified by Topp and colleagues^31^ in their systematic review as the most frequently investigated. Further, previous intervention programmes were tailored to a specific skin disease to account for differences in disease characteristics. The current study addressed stigmatisation in psoriasis.

The findings are similar to the results of previously conducted stigma interventions in chronic visible skin diseases: Interventions establishing contact between patients and the general public reported positive results.^32^, ^33^ Positive results were also found for information‐based approaches as well as approaches based on contact with affected groups to foster patient participation.^34^

However, previous stigma interventions mainly targeted patients with leprosy and were conducted in low‐ and middle‐income countries. Moreover, substantial heterogeneity in study design as well as quality was observed, making direct comparison of results—especially with regard to the interventions' effectiveness—difficult.^31^

The current results bear greatest similarity in both the methodology and the findings to interventions addressing the stigma of mental illness. Corrigan et al.^35^ report a higher effectiveness of direct contact in changing stigmatising attitudes and behaviours in adults, compared to educational approaches. The results for reported and intended behaviour are in line with studies investigating medium‐ and long‐term outcomes: No evidence of effectiveness in improving behavioural outcomes was found, but—similar to the current results for stereotype endorsement and illness‐related misconceptions—evidence of effectiveness in improving knowledge and attitudes was found.^36^

Further explanation for the lack of significant change over time as well as the lack of difference between the intervention and control groups in two of the four outcome variables might lie with the initial values: The study by Pearl et al.,^26^ which used the same assessment tools, reports higher mean scores in their laymen sample compared to the current sample. Taking the social distance values as an example, on average, scores around the value of 2.7 were observed, compared to an average of 1.78 at the beginning of the current study. Students in both groups therefore expressed a rather low wish for social distance from individuals with psoriasis from the outset, making further reduction of scores during the intervention less likely.

### 4.1 Limitations

The following limitations need to be taken into consideration when interpreting the current results: Randomised assignment to control and intervention groups was not feasible considering the structure of the vocational training. To minimise selection bias, group assignment did not depend on the researchers involved as much as it did on matches in schedules. The two groups did not differ significantly in any of the control or outcome variables at the beginning of the study.

Since the study was designed and implemented under as realistic conditions as possible, not all influencing variables could be controlled for completely. One overarching aim that guides the school's vocational training lies in the perception and understanding of human diversity and inclusion of children with, for example, disabilities. Therefore, it cannot be ruled out that the general tenor of regular classes and/or internships might have influenced the results.

Relating thereto is the sample itself: As future educators at a college of social pedagogy, students had working experience in a social profession either due to a 1‐year internship and/or their previous jobs. Taken together with their ongoing vocational education, there might have been more awareness of and familiarity with the topic of exclusion/bullying among them even though the term stigmatisation/discrimination may not have been explicitly stated in their regular classes. As indicated above, on average, stigmatising attitude scores in the sample were lower compared to the layperson sample and more similar to the sample of medical students in the study that originally used the current assessment tools,^26^ further corroborating this assumption. Future studies should therefore also include different vocational colleges to ascertain the potential influence of their curricula on stigmatising attitudes in addition to effects of the intervention. Conducting and evaluating interventions in other target groups that already are, or will be in frequent contact with individuals with skin diseases due to their occupation, like for example hair‐dressers or swimming pool attendants, would be an important addition to anti‐stigma efforts in general and the current findings in particular.

In line with this, future investigations should include other skin diseases and/or more than one skin disease to ascertain the effectiveness of the intervention beyond psoriasis. Several student remarks in the free‐text feedback option box addressed this constraint, mentioning a great interest in and wish for inclusion of different skin diseases as well as more than one patient in future seminars.

Another limitation that needs to be considered is the time frame of the current evaluation, which makes causal interpretations of the current results difficult if not impossible. While 12 weeks allow for a discussion of short‐ to midterm effects, there is still a need for longitudinal studies to properly estimate the long‐term effectiveness of the short intervention as well as potential causal associations between outcomes and control variables.

## 5 CONCLUSION

The current short intervention to reduce stigmatising attitudes showed promising effects over time as well as in comparison to the control group in future educators. Moreover, it was one of the first to evaluate a short intervention under controlled circumstances and, as such, provides proof of concept for future studies with regard to its continued optimisation and further examination of its effectiveness in various contexts and over a longer period of time. In perspective, integrating revised and adapted versions of the short intervention into the regular curricula of schools and colleges, as well as establishing these trainings for different occupational groups could help individuals with visible skin diseases in two ways: First, by preventing and/or reducing stigmatisation and discrimination in various demographics and second, by acknowledging individuals with visible skin diseases as experts on their own behalf and having them participate as an integral part of the intervention, thus further promoting patient empowerment.

## CONFLICT OF INTERESTS

The authors declare that there are no conflict of interests.

## AUTHOR CONTRIBUTIONS

Matthias Augustin, Claudia Luck‐Sikorski, Rachel Sommer and Ulrich Mrowietz conceptualised, designed and led the study. Sven M. John contributed to the study design. All authors developed the short intervention and study materials. Rachel Sommer, Sonja Mrowietz and Regina von Spreckelsen recruited participants and collected data. Natascha‐Alexandra Weinberger analysed the data. Natascha‐Alexandra Weinberger and Claudia Luck‐Sikorski interpreted the study results. Natascha‐Alexandra Weinberger and Sonja Mrowietz wrote the first manuscript. All authors discussed the results and findings and also discussed and edited the final manuscript.

## Supporting information

Supporting information.

## Data Availability

The data that support the findings of this study are available from the corresponding author upon reasonable request.
