# Supplementary material for: Effectiveness of a structured short intervention against stigmatisation in chronic visible skin diseases: Results of a controlled trial in future educators
Source: Health Expect. 2021 Jul 27;24(5):1790–800. doi: 10.1111/hex.13319 (PMC8483191; doi:10.1111/hex.13319)
Supplement: Supplementary file 1 — Supporting information. [file HEX-24-1790-s001.docx]

**Appendix**

Supplementary Table 1. Structure, content, and approximate expenditure of time for the developed short intervention.

| **Title** | **Lesson content** | **Time (min)** |
| --- | --- | --- |
| ***introduction*** | - Overview of the sequence of lesson units - Definition: „Stigma“ | 10 |
| ***self-reflection*** | - Participants reflect on situations in which they - felt stigmatized - stigmatized themselves - stigmatized others | 10 |
| ***discourse*** | - Participants discuss their experiences (description of the respective situation, their feelings and consequences) and share their ideas on the underlying processes of stigmatization | 10 |
| ***education stigmatization*** | - Lecture on stigma and the process of stigmatization and discrimination | 10 |
| ***education skin diseases*** | - Fact sheets on skin diseases are distributed (symptoms, causes, etc.) - Participants identify and discuss disease patterns with particularly stigmatizing manifestations | 20 |
| ***self-awareness exercise*** | - Participants take part in a self-awareness exercise by visibly wearing scar tattoo-stickers during lunch-break and observing reactions of other people in the cafeteria. | 20 |
| ***encounter with patient*** | - The patient affected by a chronic, visible skin disease introduces himself and shares his personal experience with stigmatization. - Participants are then able to ask questions and interact. A medical professional moderates the discussion and responds to questions e.g. regarding treatment. | 45 |
| ***self-reflection, discourse & recap*** | - Participants share and discuss their experiences during the self-awareness exercise. - Participants reflect on the previous lessons and the patient encounter, and share their personal conclusions from the seminar. | 10 |
| ***case study*** | - Participants apply the previous lesson content to case studies by identifying the underlying stigmatization process and developing first ideas to deal with or counter the specific situation. Results are presented and discussed. | 45 |
| **Total intervention** |  | 180 |
